# Reduced gray matter volume and respiratory dysfunction in Parkinson’s disease: a voxel-based morphometry study

**DOI:** 10.1186/s12883-018-1074-8

**Published:** 2018-05-26

**Authors:** Sieh-Yang Lee, Meng-Hsiang Chen, Pi-Ling Chiang, Hsiu-Ling Chen, Kun-Hsien Chou, Yueh-Cheng Chen, Chiun-Chieh Yu, Nai-Wen Tsai, Shau-Hsuan Li, Cheng-Hsien Lu, Wei-Che Lin

**Affiliations:** 1 Department of Diagnostic Radiology, Kaohsiung Chang Gung Memorial Hospital and Chang Gung University College of Medicine, 123 Ta-Pei Road, Niao-Sung, Kaohsiung, 83305 Taiwan; 2 Brain Research Center, National Yang-Ming University, Taipei, Taiwan; 3 Institute of Neuroscience, National Yang-Ming University, Taipei, Taiwan; 4 Department of Neurology, Kaohsiung Chang Gung Memorial Hospital and Chang Gung University College of Medicine, 123 Ta-Pei Road, Niao-Sung, Kaohsiung, 83305 Taiwan; 5 Department of Oncology and Hematology, Kaohsiung Chang Gung Memorial Hospital and Chang Gung University College of Medicine, Kaohsiung, Taiwan

**Keywords:** Parkinson’s disease, Magnetic resonance imaging, Gray matter, Respiratory system, Autonomic nervous system

## Abstract

**Background:**

The respiratory dysfunction of patients with Parkinson’s disease (PD) has drawn increasing attention. This study evaluated the relationship between gray matter volume (GMV), as determined by voxel-based morphometry (VBM), and respiratory dysfunction in patients with PD and correlated it with systemic inflammatory markers.

**Methods:**

Whole-brain VBM analysis was performed on 3-dimensional T1-weighted images in 25 PD patients with abnormal pulmonary function (13 men, 12 women; mean age: 62.9 ± 10.8 years) and, for comparison, on 25 sex- and age-matched PD patients with normal pulmonary function (14 men, 11 women; mean age: 62.3 ± 6.9 years). Inflammatory markers were determined by flow cytometry. The differences and correlations in regional GMV, clinical severity and inflammatory markers were determined after adjusting for age, gender and total intracranial volume (TIV).

**Results:**

Compared with the normal pulmonary function group, the abnormal pulmonary function group had smaller GMV in several brain regions, including the left parahippocampal formation, right fusiform gyrus, right cerebellum crus, and left postcentral gyri. Forced expiratory volume in 1 s (FEV1) and maximal expiratory flow after expiration of 50% of forced vital capacity (MEF50) were positively correlated with regional GMV. There were no significant differences in the level of serum inflammatory markers between two groups.

**Conclusion:**

Our findings suggested that involvement of the central autonomic network and GM loss may underlie the respiratory dysfunction in PD patients.

## Background

Respiratory dysfunction is among the leading causes of death in Parkinson’s disease (PD) [1]. The spectrum of respiratory dysfunction associated with PD includes the obstructive, restrictive, and mixed types of respiratory defects, decreased respiratory muscle strength and upper airway obstruction [2]. However, pulmonary functional impairment usually goes unnoticed until the advanced stages of the disease [3]. Therefore, the early identification and prevention of respiratory dysfunction are clinically important. Relatedly, there is accumulating evidence showing that the effects of PD on respiration occur via central and peripheral mechanisms. For example, one recent study suggested the involvement of the brain stem in the development of abnormal ventilatory control and sleep-related breathing dysfunction in PD [4], while another study found that cervical arthrosis and motor disturbances lead to upper airway obstruction and respiratory muscle impairment [3]. Despite these findings regarding respiratory disturbances, the exact pathophysiologic and anatomical brain alterations underlying respiratory dysfunction in PD have remained unclear.

The involvement of systemic oxidative stress and apoptosis in the degradation of dopaminergic neurons is emphasized in PD patients [5]. These afflicted neurons cause the deafferentiation of striatal dopamine, which eventually leads, in turn, to degeneration of the extrapyramidal system [6]. Furthermore, systemic oxidative stress has also been found to be associated with impaired pulmonary function and various lung diseases such as central obstructive pulmonary disease (COPD), asthma, and obstructive sleep apnea (OSA) [7–9]. The inflammatory markers previously found to be related to respiratory dysfunction include thiobarbituric acid reactive substances (TBARS), thiol [9], endothelial progenitor cells (EPCs) [8], soluble platelet-selectin (sP-selectin), soluble endothelial-selectin (sE-selectin), soluble intercellular (sICAM-1) and soluble vascular cell adhesion molecules (sVCAM-1) [10]. Taken together, these findings suggested a potential direct or indirect role of systemic oxidative stress in the pathogenesis of respiratory dysfunction in PD. However, the impact of systemic oxidative stress on PD patients with and without respiratory dysfunction is not yet fully elucidated.

Recent studies have demonstrated brain structural damage in certain pulmonary diseases, such as COPD and OSA [11, 12]. Meanwhile, a rat PD model revealed that brain structural deficits are related to dysfunction in respiratory rhythm generation [13]. That said, while the presence of regional gray matter changes associated with the autonomic dysfunction in PD has been well demonstrated [14], there has been little exploration of brain structural changes associated with respiratory dysfunction in PD. It is clear that the central nervous system plays a role in ventilatory regulation and the coordination of upper airway musculature [15]. A functional neuroimaging study revealed the involvement of cortical, limbic, and paralimbic (cortico-limbic) brain regions and the cerebellum in respiratory control and perception [15]. Brain structural damage in this respiratory-related cortico-limbic circuit has been found to be associated with air hunger [16] and impaired respiratory response to hypercapnia [17]. Additionally, some authors have suggested that autonomic failure might contribute to respiratory dysfunction in PD [3], while recent research has established the concept of a central autonomic network [18]. Gray matter atrophy involving part of this network has been associated with autonomic dysfunction in PD [14]. On the basis of these results and concepts, it appears that respiratory dysfunction in PD may be linked to the impairment of the cortico-limbic respiratory circuit and central autonomic network. To date, however, the relationship between respiratory dysfunction and the cortico-limbic respiratory circuit and central autonomic network has not been well demonstrated.

In this study, we aimed to evaluate the relationship between brain structural alterations, as determined by voxel-based morphometry (VBM), and respiratory dysfunction in patients with PD and to correlate them with systemic inflammatory markers. We hypothesized that PD patients with respiratory dysfunction would exhibit increased oxidative stress and regional gray matter atrophy which might be associated with impaired pulmonary function.

## Methods

### Participants

This retrospective study was approved by the local ethics committee. Written informed consent was obtained from all participants before the study. Fifty patients with diagnosis of idiopathic PD were enrolled at the Neurology Department of Kaohsiung Chang Gung Memorial Hospital. The patients were divided into two groups according to their pulmonary function test results: 25 patients were included in the abnormal pulmonary function (APF) group (13 men, 12 women; mean age: 62.9 ± 10.8 years) and 25 sex- and age-matched patients were included in the normal pulmonary function (NPF) group (14 men, 11 women; mean age: 62.3 ± 6.9 years). Two patients had a known history of cigarette smoking (one in the APF group, the other in the NPF group). The exclusion criteria included a known history of psychiatric and neurologic disorders, psychotropic medication usage, and structural abnormalities of the chest wall or a recent upper respiratory tract infection.

All the patients were diagnosed as having idiopathic PD by an experienced neurologist based on the Parkinson Disease Society’s criteria [19]. The disease severity and functional status of each patient were evaluated using the Unified Parkinson Disease Rating Scale (UPDRS), modified Hoehn and Yahr staging (H & Y) scale, and Schwab and England (S & E) activities of daily living scale during the “OFF” state.

### Pulmonary function testing

The pulmonary studies included spirometry, lung volume and pulse oximetry testing. All the pulmonary function tests were performed according to American Thoracic Society/European Respiratory Society criteria [20, 21]. Forced vital capacity (FVC), forced expiratory volume in 1 s (FEV 1), maximal expiratory flow after expiration of 50% of FVC (MEF50) and oxygen saturation (SpO2) values were measured using a spirometer (MasterScreen PFT; Jaeger, Hoechberg, Germany). All measurements of pulmonary function test were expressed as percentages of predicted normal values.

Patients were diagnosed as having central obstructive pattern with FEV1 < 80% and FEV1/FVC < 80%, peripheric obstructive pattern with MEF50 < 70%, and restrictive pattern with FVC < 80% and FEV1/FVC > 80% [3]. Patients with these abnormal pulmonary functional parameters were assigned to the APF group.

### Blood sampling and assessment of inflammatory markers

All the patients underwent blood sampling by venipuncture of forearm veins.

#### Assessment of inflammatory markers

The serum concentrations of TBARS and thiol were measured in all the patients in order to detect lipid peroxidation and determine anti-oxidative defense capability, respectively [22]. In addition, the level of EPCs was measured by flow cytometry based on a previous report [23]. First, mononuclear cells (10^6^) were incubated for 30 min at 4 °C in a dark room with monoclonal antibodies against kinase insert domain-conjugating receptor (KDR) (Miltenyi Biotec, Bergisch Gladbach, Germany) and fluorescein isothiocyanate-conjugated CD34 and CD133, by which the EPC surface markers of CD133/CD34 and KDR/CD34 were determined. The control ligand (IgG-fluorescein isothiocyanate conjugate) was then added. Quantitative two-color flow cytometric analysis was performed using an Epics XL flow cytometer (Beckman Coulter). In these arrays, each analysis included 10,000 cells per sample and was performed in duplicate, with mean level reported.

#### Assessment of serum adhesion molecules

To assess serum sICAM-1, sE-selectin, and sP-selectin levels, commercially available enzyme-linked immunosorbent assays (R & D Systems, Minneapolis, MN, USA) were used [24]. The dual wavelength absorbance, from which the degree of enzymatic turnover of the substrate was estimated, was measured at 450 and 620 nm. Absorbance was directly proportional to the concentration of antigens present. To determine the antigen concentrations of the unknowns, a standard curve of absorbance of standard antigen versus the given antigen concentration was plotted.

### MR imaging

#### Data acquisition

A GE Signa 3 T whole-body MRI scanner (General Electric Healthcare, Milwaukee, WI) using an 8-channel phase array head coil was used to perform the volumetric structural MRI scans. With 110 contiguous axial slices aligned to the anterior and posterior commissure, whole-brain 3-dimensional T1-weighted images of all participants were collected using an axial inversion-recovery prepared fast-spoiled gradient-recalled echo pulse sequence. The scanning parameters were as follows: repetition time = 9.5 ms, echo time = 3.9 ms; inversion time = 450 ms, flip angle = 15^o^; number of excitations = 1; field of view = 240 × 240 mm^2^; matrix size = 512 × 512; and voxel size = 0.47 × 0.47 × 1.3 mm^3^ (without inter-slice gap and interpolation).

#### Voxel-based morphometry analysis

Voxel-based morphometry analysis was performed using the Statistical Parametric Mapping software (SPM12 version 7219, Wellcome Institute of Neurology, University College London, UK, http://www.fil.ion.ucl.ac.uk/spm/) and Matlab R2010a (Mathworks, Natick, MA). The default settings were used unless otherwise specified. First, whole brain T1-weighted images were bias-corrected and segmented into gray matter (GM), white matter (WM), and cerebrospinal fluid using the New Segment Toolbox of SPM12. Then, the GM images were rigid aligned to the tissue probability maps in the Montreal Neurological Institute (MNI) standard space and averaged to create the study-specific tissue template using the high dimensional Diffeomorphic Anatomical Registration Exponentiated Lie (DARTEL) algorithm [25]. Subsequently, all native space GM images were registered to this study-specific template and further spatially normalized into standard MNI space (1.5 mm isotropic voxel). The resulting GM images were modulated by Jacobian determinant of the corresponded deformation filed to correct for volume changes. Finally, the modulated GM images were smoothed using an isotropic Gaussian kernel of 8 mm full-width at half maximum.

### Statistical analysis

#### Analysis of demographic data, pulmonary function parameters, global brain volumes, and inflammatory markers

All statistical analyses of demographic data and global tissue volumes were performed using SPSS software, version 22, for Windows (SPSS, Chicago, IL). Age and sex were compared between groups by the 2-sample Student t test and Pearson chi-squared test, respectively. Analysis of covariance (ANCOVA) was used to analyze differences in pulmonary functional parameters, clinical severity, inflammatory markers, and global brain volume with the participant’s age and sex as covariates. All data were reported as the mean ± standard deviation (SD). The *P* value for statistical significance was set at < 0.05.

#### Analysis of between-group regional GMV differences

To examine between-group differences in regional GM volume, a voxel-wise general linear model was used to compare GM volume between APF group and NPF group using 1-factor 2-level ANCOVA design with age, sex and total intracranial volume as covariates. The statistic threshold was set at cluster-level family-wise error (FWE) corrected *P*-value< 0.05, with a cluster size of at least 287 voxels, based on the results of a Monte Carlo simulation using the command-line tool of Analysis of Functional NeuroImages software (AFNI; Version AFNI_17.1.04; http://afni.nimh.nih.gov/afni/; 3dClusterSim with the following parameters: voxel *P*-value< 0.005, with explicit GM mask and 10,000 simulations). The regional GM volume of clusters with significant between-group differences were extracted and averaged for further correlation analysis.

#### Correlations between regional GMVs and pulmonary functional parameters

Partial correlation analysis was performed to correlate the pulmonary functional parameters with regional GMs of reduced volumes, inflammatory parameters, and disease severity after controlling for age, sex and TIV. The threshold for statistical significance was set at *P*-value < 0.05 with Bonferroni correction for multiple comparisons.

## Results

### Demographic characteristics of the participants

As listed in Table 1, the APF group and NPF group had similar mean ages and sex distributions (Age: *p* = 0.828 and sex: *p* = 0.777). There were also no significant differences in clinical severity between the two groups.

**Table 1:** Demographic and Clinical Data of PD Patients in APF Group and NPF Group

	Patients with PD		
Clinical demographics	APF group, *n* = 25	NPF group, *n* = 25	*P*-value
Age (years)	63 ± 11	62 ± 7	0.828
Sex (male/female)	13/12	14/11	0.777
GMV	546.0 ± 75.0	578.6 ± 57.8	0.098
WMV	432.1 ± 57.3	432.6 ± 45.4	0.871
TIV	1337.6 ± 134.4	1364.6 ± 133.7	0.441
Pulmonary function parameters			
FVC (% pred)	85.4 ± 20.4	99.8 ± 13.6	0.030^*^
FEV1 (% pred)	84.1 ± 19.1	105.9 ± 14.0	< 0.001^*^
FEV1/FVC	79.7 ± 9.0	86.7 ± 4.9	< 0.001^*^
MEF 50 (%)	63.2 ± 22.9	98.7 ± 14.2	< 0.001^*^
SpO2 (%)	94.5 ± 3.4	94.3 ± 2.7	0.756
Disease severity scale			
UPDRS I	4.1 ± 3.6	3.3 ± 2.5	0.435
UPDRS II	12.5 ± 9.7	9.7 ± 5.6	0.311
UPDRS III	28.8 ± 23.0	23.5 ± 11.8	0.396
UPDRS total	45.4 ± 35.1	36.5 ± 18.8	0.357
Modified H & Y	2.2 ± 1.3	1.8 ± 0.9	0.276
S & E	78.3 ± 20.8	86.7 ± 11.1	0.128
Inflammatory markers			
TBARS (*µ*M/L)	13.9 ± 5.1	13.1 ± 5.1	0.520
Thiol (*µ*M/L)	1.3 ± 0.5	1.5 ± 0.4	0.360
ICAM-1	215.1 ± 65.6	195.3 ± 75.8	0.514
P-selectin	100.5 ± 18.1	97.0 ± 13.0	0.314
E-selectin	42.6 ± 18.3	34.0 ± 18.0	0.243
CD133^+^CD34^+^ (%)	18.4 ± 16.1	27.7 ± 27.9	0.195
KDR^+^CD34^+^ (%)	3.5 ± 4.5	3.2 ± 4.7	0.943

*APF* abnormal pulmonary function, *EPC* epithelial progenitor cell, *F* female, *FVC* forced vital capacity, *FEV1* forced expiratory volume in 1 s, *GMV* gray matter volume, *ICAM-1* intercellular adhesion molecule 1, *KDR* kinase insert domain-conjugating receptor, *M* male, *MEF50* maximal expiratory flow after expiration of 50% of FVC, *modified H & Y* modified Hoehn and Yahr staging scale, *NPF* normal pulmonary function, *PD* Parkinson’s disease, *SpO2* oxygen saturation, *S & E* Schwab and England Activities of Daily Living Scale, *TIV* total intracranial volume, *TBARS* thiobarbituric acid reactive substances, *UPDRS* Unified Parkinson Disease Rating Scale, *WMV* white matter volume

All data are presented as mean ± standard deviation. ^*^P < 0.05

### Differences in pulmonary function test results between groups

The pulmonary functional parameters of the participants are shown in Table 1. The APF group had significantly lower FEV1, FVC, FEV1/FVC, and MEF50 values than the NPF group.

### Differences in regional GMV loss between groups

**Fig. 1:**
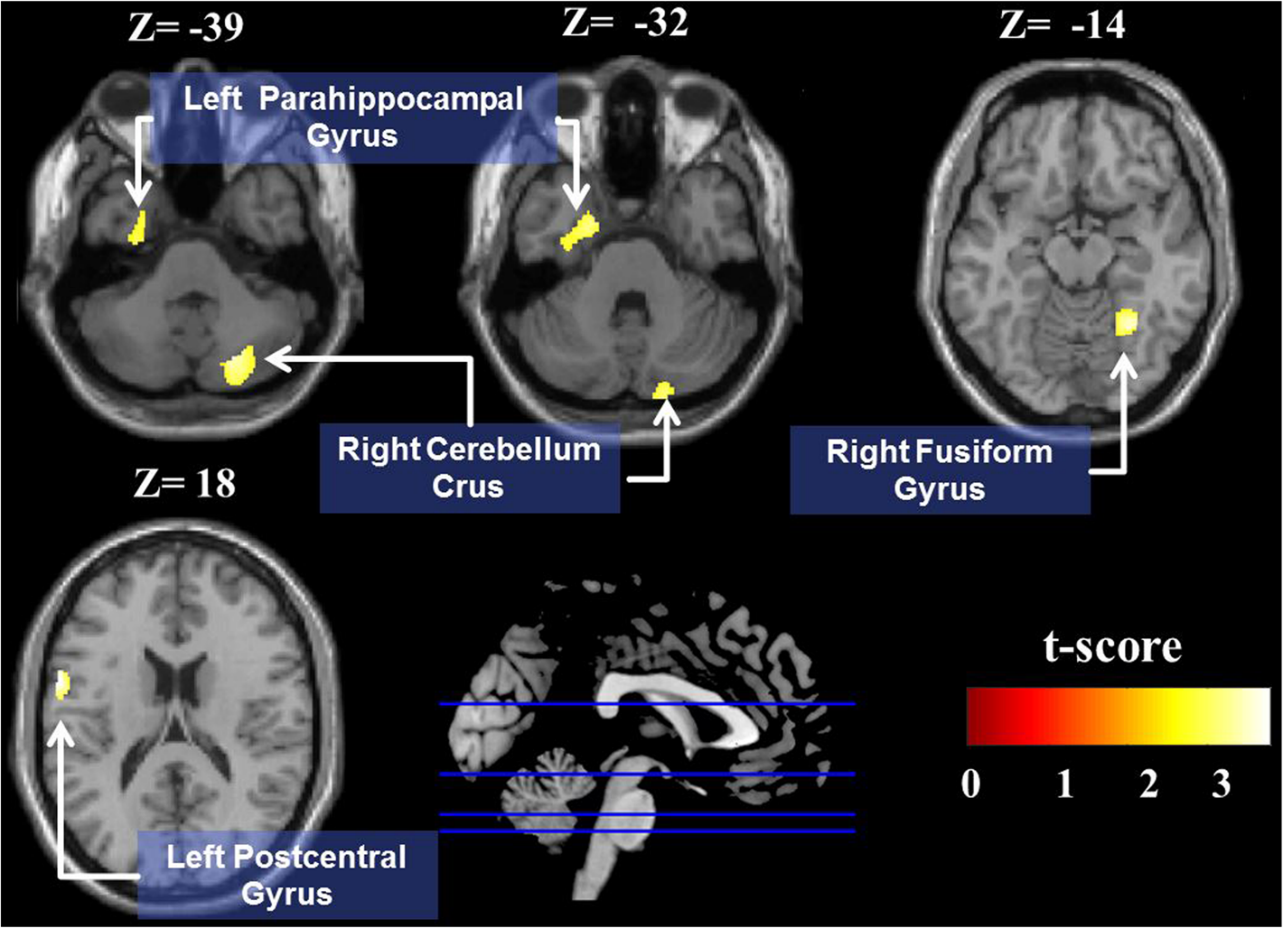
Voxel-based morphometry analysis between APF group and NPF group. Smaller GMVs in APF group versus NPF group in the left parahippocampal formation, right fusiform gyrus, right cerebellum crus, and left postcentral gyri (FWE corrected *P* value < 0.05)

As shown in Fig. 1 and Table 2, the APF group had significantly smaller GMVs in the left parahippocampal gyrus, right fusiform gyrus, right cerebellum crus, and left postcentral gyri than the NPF group.

**Table 2:** Regions Showing GMV Differences between APF Group and NPF Group

	MNI coordinates				GMV, mm3		
Region	x	y	z	Voxel size	APF group	NPF group	tmax
Postcentral gyrus, L	−60	3	18	421	333.19 ± 46.97	388.16 ± 52.92	3.79
Fusiform gyrus, R	30	−54	−14	494	492.38 ± 64.35	546.86 ± 57.47	3.57
Cerebellum crus, R	23	−74	−39	723	523.49 ± 86.86	599.08 ± 72.40	3.43
Parahippocampal gyrus, L	−20	−2	−32	663	483.20 ± 74.66	547.60 ± 69.73	3.17

*APF* abnormal pulmonary function, *GMV* gray matter volume, *L* left, *MNI* Montreal Neurological Institute, *NPF* normal pulmonary function, *PD* Parkinson’s disease, *R* right

The statistical criteria are presented with voxel height uncorrected *P* < 0.001 and a cluster extent threshold of the family-wise error rate (FWE) < 0.05

### Differences in inflammatory markers between groups

There were no significant differences in the level of TBARS, level of thiol, circulating EPCs (CD133/CD34 and KDR/CD34) and serum adhesion molecules (sICAM-1, sE-selectin, and sP-selectin) between the two groups (Table 1).

### Correlations between pulmonary functional parameters and GM volumes

**Fig. 2:**
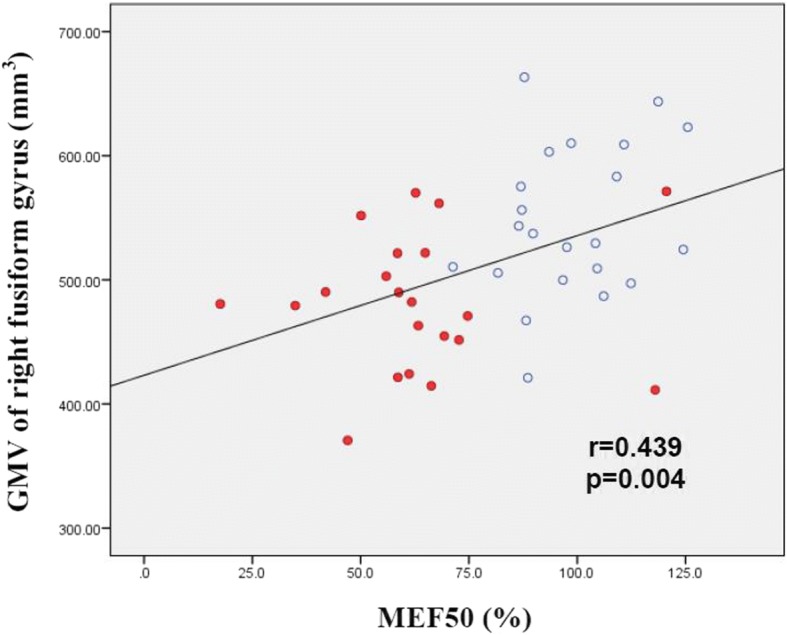
Scatter plot of GMV versus MEF50 for patients in APF group (red dots) and NPF group (open circles). Smaller GMV in the right fusiform gyrus was associated with lower pulmonary functional parameters (i.e., MEF50 [*r* = 0.439, *p* = 0.004])

In the APF group, the partial correlation analysis showed that lower MEF50 values were associated with smaller GMVs of the right fusiform gyrus (*r* = 0.439, *p* = 0.004) (Fig. 2), right cerebellum crus (*r* = 0.314, *p* = 0.046), and left postcentral gyri (*r* = 0.339, *p* = 0.030) after controlling for sex, age and TIV. Using a Bonferroni-correlated *p* value < 0.05, multiple comparisons revealed a significant correlation between the right fusiform gyrus GMV and MEF50. Additionally, lower FEV1 values were also associated with smaller GMVs of the right fusiform gyrus (*r* = 0.359, *p* = 0.021) and right cerebellum crus (*r* = 0.370, *p* = 0.017). FVC and FEV1/FVC values had no significant correlations with GMV (Table 3).

**Table 3:** Correlations between Pulmonary Functional Parameters and Gray Matter Volume

	Pulmonary functional parameters			
GMV anatomical location	FEV1 (%, pred)	FVC (%, pred)	FEV1/FVC	MEF 50 (%)
Postcentral gyrus, L	0.228	0.085	0.200	0.339^*^
Fusiform gyrus, R	0.359^*^	0.268	0.169	0.439^**^
Cerebellum crus, R	0.370^*^	0.240	0.207	0.314^*^
Parahippocampal gyrus, L	0.194	0.125	0.138	0.282

*FVC* forced vital capacity, *FEV1* forced expiratory volume in 1 s, *GMV* gray matter volume, *L* left, *MEF50* maximal expiratory flow after expiration of 50% of FVC, *R* right. ^*^P < 0.05, ^**^P < 0.005

## Discussion

Using VBM analysis, this study demonstrated the presence of brain structural alteration in PD patients with respiratory dysfunction. Consistent with our hypothesis, the APF group showed smaller GMVs in several brain regions than the NPF group. Furthermore, GMV loss in these brain regions was specifically correlated with poor pulmonary parameters as evaluated by pulmonary function tests. This is the first study to establish an association between respiratory dysfunction and regional cortical deficits in PD.

According to the Braak staging hypothesis [26], the damage to the brainstem occurs earliest in PD. The early involvement of the medullary respiratory center might account for the autonomic dysfunction and respiratory dysfunction in the early stage of the disease [4, 27]. However, the various phenotypes of respiratory dysfunction in PD cannot be attributed solely to the involvement of the brainstem. Interestingly, our present study showed that the APF group had smaller GMVs in some parts of the central autonomic network, including the cerebellum crus, parahippocampal gyrus, and fusiform gyrus. It has previously been recognized that the autonomic nervous system plays a pivotal role in the regulation of lung ventilation, gas exchange, and airway smooth muscle [28]. Moreover, growing evidence suggests that the central autonomic network participates in regulatory control of sympathetic, parasympathetic, and respiratory motor neurons [29]. In the proposed concept of the central autonomic network [18], sympathetic regulation is primarily associated with the cingulate gyrus, cerebellum, insula, and supplementary motor area, whereas parasympathetic regulation is associated with the hippocampal formation, amygdala, insula, and cerebellum. The association of regional cortical deficits with autonomic dysfunction [14] supported our hypothesis that interference with the central autonomic network in PD might contribute to the development of respiratory dysfunction.

Our results also showed that the smaller GMVs in several brain regions of the APF group were positively correlated with pulmonary functional parameters, particularly FEV1 and MEF50. FEV1 and MEF50 are markers used to assess airway obstruction and detect early small airway diseases such as COPD and asthma in clinical practice [30]. These alterations might be explained by the complex sympathetic and vagal innervation of the lung. The autonomic nerve supply is one of the main mechanisms controlling the contraction of the airway smooth muscle [28]. The vagus causes bronchoconstriction and increased airway resistance, while the sympathetic nerves are commonly thought to cause the relaxation of bronchial caliber and diminished airflow resistance [28]. Although the mechanism remains unclear, it seems that gray matter deficits in PD would interfere with the autonomic innervation of the airways, leading to increased airflow resistance and airway obstruction.

Furthermore, compared to the NPF group, the APF group had smaller GMVs in the cerebellum, parahippocampal gyrus, and fusiform gyrus, regions which are considered to be principal components of respiratory-related cortico-limbic circuitry. In the proposed model for respiratory sensorimotor neural circuitry [15], the motor division comprises the motor cortex, supplementary motor area, basal ganglia, cerebellum, and brainstem, while the sensory division includes the insula, amygdala, sensory cortex, and cerebellum. The motor division is responsible for volitional breathing via cortico-spinal circuitry, whereas the sensory division mediates the integration of processing of dyspnea stimuli. The neuronal activation within the parahippocampal gyrus, fusiform gyrus, and parietal cortex during CO2-stimulated breathing demonstrate the regulatory capacity of supra-brainstem structures in sensory and motor respiratory responses to hypercapnia [17]. Our results are highly consistent with the physiological findings of the previous studies.

The cerebellum is classically associated with the regulation of motor coordination. However, the roles of cerebellar structures in respiratory patterns [16] and autonomic dysfunction [14] are also well recognized. The involvement of the cerebellum in the experience of hypercapnia and air hunger has been established [16]. Moreover, the parahippocampal gyrus and fusiform gyrus, traditionally thought to be involved in memory and cognitive functions, also participate in normal respiratory responses to chemosensory stimuli [17]. Thus, cortical deficits in the cerebellum, fusiform gyrus, and parahippocampus may explain abnormal ventilatory responses to CO2 in PD [27]. These findings further support the assumption that GMV loss might play contributory roles in the development of respiratory dysfunction in PD.

Elevated systemic oxidative stress has been demonstrated to be the main etiology of PD [5]. The imbalance between oxidative stress and antioxidative capacity results in elevated neuroinflammation, the death of dopaminergic neurons, and a cascade of cell repairing processes. In recent studies, alterations to systemic oxidative stress have also been found to have profound associations with various lung diseases [7–9] and impaired pulmonary functional parameters [9, 10]. Although previous studies have yielded relevant associations, in this study, the APF group showed no significant differences in systemic oxidative stress with the NPF group, presumably because of the small sample of recruited patients. However, while the results of the study cannot clarify the influences of oxidative stress on respiratory dysfunction in PD, they do allow us to draw a direct association between brain structural alterations and pulmonary function impairments with less concern over potential confounding factors such as oxidative stress and clinical severity.

### Limitations

While significant results were found with respect to brain structural alterations, several limitations of our study should be acknowledged. First, our results may have been influenced by the lack of a healthy control group and the relatively small sample size, which may explain the insignificant differences for GMV among the APF subgroups. Second, the complex mechanism underlying the oxidant/antioxidant balance of PD patients may be affected by various factors, including individual genetic variations and physical exercise, and these confounding factors were not comprehensively addressed in our study. Finally, the final sample of 25 PD patients in the APF group did not allow division of the group into subgroups in order to further examine relevant variables. Although no significant GMV differences were found among the different types of respiratory dysfunction in this study, we cannot exclude the possibility that brain structural alterations associated with specific types of respiratory dysfunction could be detected with a larger sample. Future longitudinal studies utilizing larger sample sizes and including healthy controls could help to better characterize the different phenotypes and clarify the causal relationship underlying respiratory dysfunction in PD.

## Conclusions

This study is the first to demonstrate that respiratory dysfunction in PD patients is associated with gray matter loss in specific brain regions. Our findings suggest significant participation on the part of the central autonomic network and the contribution of gray matter loss to the development of respiratory dysfunction in PD. These results give us a deeper insight into the pathophysiological basis of respiratory dysfunction in PD.

## Abbreviations

APF: Abnormal pulmonary function
COPD: Chronic obstructive pulmonary disease
EPC: Epithelial progenitor cell
E-selectin: Endothelial selectin
FEV1: Forced expiratory volume in 1 s
FVC: Forced vital capacity
GMV: Gray matter volume
ICAM-1: Intercellular adhesion molecule 1
KDR: Kinase insert domain-conjugating receptor
MEF50: Maximal expiratory flow after expiration of 50% of FVC
modified H & Y: Modified Hoehn and Yahr staging
NPF: Normal pulmonary function
OSA: Obstructive sleep apnea
PD: Parkinson’s disease
P-selectin: Platelet-selectin
S & E: Schwab and England activities of daily living
TBARS: Thiobarbituric acid reactive substances
TIV: Total intracranial volume
UPDRS: Unified Parkinson Disease Rating Scale
VBM: Voxel-based morphometry
VCAM-1: Vascular cell adhesion vascular 1
WMV: White matter volume
